# Development and Validation of a Clinical Trial Patient Stratification Assay That Interrogates 27 Mutation Sites in MAPK Pathway Genes

**DOI:** 10.1371/journal.pone.0072239

**Published:** 2013-08-21

**Authors:** Ken C. N. Chang, Stefan Galuska, Russell Weiner, Matthew J. Marton

**Affiliations:** Clinical Development Laboratory, Merck & Co, Inc., Rahway, New Jersey, United States of America; University of Texas MD Anderson Cancer Center, United States of America

## Abstract

Somatic mutations identified on genes related to the cancer-developing signaling pathways have drawn attention in the field of personalized medicine in recent years. Treatments developed to target a specific signaling pathway may not be effective when tumor activating mutations occur downstream of the target and bypass the targeted mechanism. For instance, mutations detected in *KRAS/BRAF/NRAS* genes can lead to EGFR-independent intracellular signaling pathway activation. Most patients with these mutations do not respond well to anti-EGFR treatment. In an effort to detect various mutations in FFPE tissue samples among multiple solid tumor types for patient stratification many mutation assays were evaluated. Since there were more than 30 specific mutations among three targeted *RAS/RAF* oncogenes that could activate MAPK pathway genes, a custom designed Single Nucleotide Primer Extension (SNPE) multiplexing mutation assay was developed and analytically validated as a clinical trial assay. Throughout the process of developing and validating the assay we overcame many technical challenges which include: the designing of PCR primers for FFPE tumor tissue samples versus normal blood samples, designing of probes for detecting consecutive nucleotide double mutations, the kinetics and thermodynamics aspects of probes competition among themselves and against target PCR templates, as well as validating an assay when positive control tumor tissue or cell lines with specific mutations are not available. We used Next Generation sequencing to resolve discordant calls between the SNPE mutation assay and Sanger sequencing. We also applied a triplicate rule to reduce potential false positives and false negatives, and proposed special considerations including pre-define a cut-off percentage for detecting very low mutant copies in the wild-type DNA background.

## Introduction

Mutational status of solid tumors is becoming increasingly important for identifying the best treatment options for cancer patients [1]. Treatments developed against a specific signaling pathway may not be effective when activating mutations are downstream of the signal transduction pathway. For example, tumors harboring activating mutations in *RAS* and *RAF* do not respond well to anti-EGFR therapy, since these mutations can lead to EGFR-independent activation of intracellular signaling pathway [2]. Alternatively, inhibitors against proteins further downstream in the pathway may be more effective. Proteins that lie downstream of the small guanosine triphosphatase (GTPase) *RAS* and the protein kinases *RAF* and *MEK* in the *RAS*/mitogen-activated protein kinase (MAPK) pathway are potential targets for pharmacological intervention [3]–[5]. Gain of function, i.e. activating, mutations in *RAS* and *RAF* resulting in constitutive activation of this pathway are frequently observed in human cancers and are associated with high rates of cancer cell proliferation [3]–[5]. It has been reported that activating mutations of *RAS* are identified in ∼25% of all cancers [6]. These mutations, especially in *KRAS*, are present at even higher rates in pancreatic cancer and colorectal cancer [3], [6]. Other studies also reported that *NRAS* mutations were detected in ∼10–25% of melanomas [3], [5]–[7] and *KRAS* mutations were detected in ∼30% non-small cell lung cancers (NSCLCs) [3], [8]. In addition, *RAS* mutations (*HRAS*, *KRAS*, or *NRAS*) have been identified in ∼55–60% of thyroid cancers, and *BRAF* mutations have been identified in ∼60% of malignant melanomas [5], [9], [10]. These *BRAF* mutations are within the kinase domain and a single substitution (T A, V600E) accounts for ∼80% of mutations [5], . Activating *BRAF* mutations have also been documented in a variety of human cancers other than melanoma, such as ∼10% in colorectal cancer (CRC), approximately 50% in thyroid cancer [7], and several percent in NSCLC [11]. The high frequency of *RAS* or *BRAF* mutations in these cancers makes targeting this pathway an attractive strategy for new anti-cancer agent development that relies on a patient stratification to identify individuals most likely to benefit from MAPK inhibitors [12].

Scientific and clinical attention is mostly focused on the major mutational hotspots in these genes (*KRAS* codons 12, 13 and 61 and *BRAF* 600). There is increasing evidence that mutations in other locations can also lead to the tumorigenic phenotype [13], [14]. We identified 35 mutations on *KRAS/BRAF/NRAS* genes that are thought to activate the MAPK pathway or increase activity of one of the kinases 15–20. Thus, there is a need for highly sensitive and specific assays to detect these mutations, initially as a patient selection clinical trial assay but ultimately as a potential companion diagnostic assay that would direct treatment in clinical practice. Sanger sequencing, the “gold standard” for detecting mutations, was ruled out as being too insensitive in FFPE tissue to detect mutations present in low amounts relative to wild type DNA. Commercially available FDA-cleared *BRAF* and *KRAS* kits, such as the Qiagen Therascreen *KRAS* RGQ kit and the Cobas *KRAS* and *BRAF* mutation tests detect mutations only at limited locations and do not include the set of mutations we are interested in. Therascreen *KRAS* assay is based on Scorpions and ARMS technologies and detects 7 frequently encountered mutations in codon 12 and 13. The Cobas *KRAS* mutation kit is a TaqMelt PCR assay which detects 21 mutations in codons 12, 13, and 61. However, specific mutations are not reported, which is important for exploratory data analysis. Cobas *BRAF* assay only detects *BRAF* V600E mutation. There is no commercial *NRAS* mutation detection assay. A PCR-based approach to detect all these mutations would likely require more than 100 primer/probe sets and at least 30 individual reactions. Several qualitative mutant-enriched PCR assays appear to be very sensitive in detecting low mutant copies such as commercially available StripAssay [21]. However in addition to the large number of assays needed to be designed to cover all desired mutations, no percent mutant for each mutation in the corresponding samples could be estimated as only yes or no answers are obtained. Since treatment responses may be directly related to a particular mutation, retrospective analysis of such information could lead to hypotheses to better predict patient treatment response. Because of the lack of viable options, we developed and validated an efficient semi-quantitative multiplexed assay called KBN-SNPE (*KRAS/BRAF/NRAS* SNPE assay) to detect a broader array of mutations. Similar assays were used in several different applications for clinical sample analysis [22]–[24], however none were specifically designed and validated to prospectively enroll patients for an on-going clinical trial.

## Materials and Methods

### Cell Lines and Formalin Fixed Paraffin Embedded (FFPE) Samples

All cell lines were obtained from ATCC and cultivated using recommended conditions. Genomic DNA (gDNA) was extracted from cell pellets using DNeasy (Qiagen, Valencia, CA). Sixty Formalin Fixed Paraffin Embedded (FFPE) samples, ten FFPE slides each, from the following cases of human cancers were purchased from BioChain (Newark, CA): colorectal, melanoma, ovarian, thyroid, pancreatic, and lung (non-small cell lung cancers). Tissue samples were immediately fixed in formalin and paraffin embedded. Five micron thick sections were cut and mounted on positively charged slides. FFPE samples were shipped at room temperature and stored at 4–8°C. Extraction of gDNA from FFPE tissue samples was performed using the Qiagen DNA FFPE Tissue Extraction Kit (Qiagen, Valencia, CA) following manufacturer’s instructions. Briefly, the excess paraffin was removed from one or two 5 micron thick sections and the tissue scraped off the slide using a fresh surgical scalpel. A new scalpel was used for each section. The paraffin was removed by successive washes with xylene and ethanol and the tissue digested with proteinase K. The released gDNA was bound and eluted from a miniElute column in 50 µL, quantified using the Nanodrop and adjusted to a concentration of 5 ng/uL and stored at −80°C.

### Ethics Statement

All FFPE samples of human cancer tissues were obtained from Biochain. BioChain’s tissue products are based on the sample repository network established following the IRB-approved ethical standard and procedures.

### PCR Primers and Single Nucleotide Primer Extension (SNPE) Probes and Synthetic Oligonucleotides

PCR primers (Table S1), SNPE probes (Table S2) and synthetic oligonucleotides (Table S3) were obtained from Sigma-Aldrich Corp (St. Louis, MO) and were either desalted or purified by HPLC. Except where noted, all reagents for PCR and SNPE reactions were obtained from Life Technologies (Carlsbad, CA). PCR primers were designed to amplify gene fragments ranging in size from 100–150 nucleotides of the appropriate regions of *KRAS*, *NRAS* and *BRAF*. To accomplish this, DNA sequences surrounding the regions of interest were manually scanned to identify portions which could serve as primers to amplify the appropriate sized fragment of DNA (Figure S1). BLAST searches with the amplified regions were performed to confirm specificity of the PCR amplified product. Since individual exons were not equally amplified during the PCR amplification step, it was necessary to systematically adjust the concentration of the various PCR primers until all exons were equally amplified.

Probes for the SNPE reaction were chosen by selecting regions of genes immediately adjacent 3′ to the base of interest. Both sense and antisense strands were used in designing SNPE probes. Size based resolution of the probes was made possible by the addition of GATC repeats or poly T tails (GATC, T10 up to T49 in Table S2) on the 5′ end of the probe. In one case a mixed poly T-C oligo tail (T10 C10 T36) was used to circumvent problems synthesizing a poly T56 nucleotide tail.

### Single Nucleotide Primer Extension (SNPE) Assay

Fifteen (15) ng of isolated gDNA was used as template for PCR amplification of *KRAS* exons 2, 3 and 4, *NRAS* exons 2 and 3 and *BRAF* exons 11 and 15. Each gene was independently amplified by PCR. For each PCR reaction 25 µL ABI AmpliTaq Gold Master Mix (ABI cat # 4398886), 5 µL PCR primer pool (Table S2), 3 µL gDNA (5 ng/µL), and 17 µL distilled water were mixed and subjected to 40 rounds of PCR amplification. PCR conditions were as follows: 96°C, 5 minutes, 40 cycles of amplification, each cycle consists of 94°C, 30 seconds, 55°C, 55 seconds, 72°C, 45 seconds. After 40 cycles the reactions were incubated at 72°C for 10 minutes then held at 4°C until further processed for the single nucleotide primer extension (SNPE) assay. Any remaining PCR product was stored at −20°C.

Prior to using PCR product as a template in the single SNPE assay, 15 µL of each PCR product was treated with 5 units of calf intestine alkaline phosphatase (CIP, New England BioLabs, Ipswich, MA) and 2 units of Exonuclease I (ExoI, USB, Cleveland, OH) at 37°C, 1 hour, followed by 70°C, 0.25 hour in a final volume of 16 µL. 3 µL of CIP/Exo I treated PCR product was then used in the SNPE assay without further adjustment to the DNA concentration. Each single nucleotide primer extension reaction consisted of 5 µL SNaPshot Multiplex Kit (ABI), 3 µL CIP/ExoI treated PCR product, 1 µL SNPE probe pool (see Table S2 for probe sequence and concentrations used), and 1 µL water. SNPE reaction cycling conditions were as follows: 96°C, 10 seconds, 50°C, 5 seconds, 60°C, 30 seconds, for 25 cycles. Samples were maintained at 4°C until analyzed. For analysis, each SNPE reaction was treated with 1 unit CIP, 37°C, 1 hour followed by 70°C, 0.25 hour. One-half microliter (0.5 µL) of the CIP treated single SNPE reaction product was mixed with 0.5 µL GeneScan 120 LIZ size standards (ABI) and 9.0 µL Hi-Di Formamide (ABI), denatured at 95°C for 5 minutes. After denaturing the samples were immediately placed on ice for 5 minutes then centrifuged and loaded onto an ABI 3500 Genetic analyzer equipped with a 50 cm capillary using POP 7 polymer (ABI). Results of the capillary electrophoresis run were imported into GeneMapper (ABI, version 4.1) where pre-programmed base calling functions for *KRAS*, *BRAF* and *NRAS* identified the bases found at each position examined.

### Synthetic Oligonucleotides as Templates for the SNPE Assay

Synthetic oligonucleotides (Table S3), made up as 100 µM stocks in distilled water, were diluted 1∶200 in 1X CIP buffer. 15 µL of diluted stock was transferred to microtiter plate and treated with 5 units CIP at 37°C for 60 minutes followed by 70°C for 15 minutes. One microliter of the CIP treated oligonucleotide was mixed with 9 µL SNPE reaction mixture. The remaining incubation and analysis methods were the same as that described for gDNA.

### Assaying Samples in Triplicate

In experiments where samples were assayed in triplicate starting from same pool of gDNA, the rules for determining the consensus call are presented in (Table 1). If the same call is determined in 2 of 3 or 3 of 3 reactions, then the majority call is the consensus. Where a call is not possible (ND) in two or more replicates, the consensus call is considered to be indeterminate. When performed in support of a clinical trial re-do rules would be applied and samples where clear results could not be obtained would be re-assayed. Two failed attempts would result in a “No-Call” result and a new sample would be requested.

**Table 1:** Rules for calling *KBN*-SNPE results when assaying samples in triplicate.

Replicate/Call			
1	2	3	Consensus
Wild Type	Wild Type	Wild Type	Wild Type
Wild Type	Wild Type	Mutation-1	Wild Type
Wild Type	Mutation-1	Mutation-1	Mutation-1
Mutation-1	Mutation-1	Mutation-1	Mutation-1
Wild Type	Wild Type	ND*	Wild Type
Mutation-1	Mutation-1	ND	Mutation-1
Wild Type	ND	ND	Indeterminate-Redo
ND	ND	ND	Indeterminate-Redo
Mutation-1	ND	ND	Indeterminate-Redo
Wild Type	Mutation-1	ND	Indeterminate-Redo
Mutation-1	Mutation-1	Mutation-2	Mutation-1

* ND = Not Determined.

### Sanger Sequencing

Sanger sequencing was accomplished using ABI BigDye Direct Cycle Sequencing Kit (ABI # 4458687) with M13 modified PCR primers and following the manufacturer’s instructions. The reaction mixture was applied to an ABI 3500 Genetic analyzer equipped with a 50 cm capillary using POP 7 polymer (ABI).

### Ion Semiconductor Sequencing

Eighty ng of gDNA from discordant samples were analyzed by ion semiconductor sequencing on the Ion PGM sequencer (Life Technologies, Carlsbad, CA). Targeted exon enrichment was performed using the GeneRead DNA panel. Enrichment was accomplished using 20–25 PCR cycles. Library construction was made using Life Tech’s Ion Xpres Plus gDNA and Amplicon Library construction kit. Variant calls were made using two algorithms, Ion Torrent Variant Caller and GATK. Only variants identified by both algorithms were reported.

### Calculation of Specificity, Sensitivity and Concordance

Concordance was determined at the sample (patient) level. Each sample was subjected to mutation analysis by the KBN*-*SNPE assay and the Sanger sequencing as described above. If the two methods agreed, the result is assumed to be ‘truth’. If only one method detects a mutation, the sample was analyzed by Ion PGM sequencing. If the Ion PGM result matched either the KBN-SNPE or Sanger result, the matched result was considered ‘truth’ and was considered the consensus call. Performance of the KBN-SNPE assay is measured vs. this consensus call. Samples where a clear result could not be obtained were not included in the concordance calculations.

### Sample-Level Negative Agreement (Specificity)

The specificity of the KBN-SNPE assay is a measure of its ability to identify samples that are wild type for *KRAS*, *BRAF* and *NRAS*. A wild type result in a test with high specificity indicates a high probability of the mutation being absent. Specificity is defined as follows:

Definitions:

Truth: the consensus result obtained by two or more assay methods (KBN-SNPE, Sanger sequencing, NGS)

True positive = sample correctly identified as having a mutation

False positive = sample incorrectly identified as having a mutation

True negative = sample correctly identified as not having any mutations (wild type) listed in Table 2.

**Table 2:** Mutations identified by the *KBN*-SNPE assay and their resulting amino acid change.

BRAF				KRAS				NRAS			
CodonNumber	WTCodon	MutantCodon	ProteinDescription	CodonNumber	WTCodon	MutantCodon	ProteinDescription	CodonNumber	WTCodon	MutantCodon	ProteinDescription
466	GGA	GAA	G466E	12	GGT	AGT	G12S	12	GGT	GAT	G12D
466	GGA	GTA	G466V	12	GGT	TGT	G12C	12	GGT	GTT	G12V
592	ATA	GTA	I592V	12	GGT	GAT	G12D	13	GGT	CGT	G13R
594	GAT	GTT	D594V	12	GGT	GCT	G12A	13	GGT	GAT	G13D
594	GAT	GAA	D594E	12	GGT	GTT	G12V	61	CAA	AAA	Q61K
594	GAT	GAG	D594E	13	GGC	CGC	G13R	61	CAA	CGA	Q61R
596	GGT	CGT	G596R	13	GGC	GAC	G13D	61	CAA	CTA	Q61L
597	CTA	TCA	L597S	61	CAA	AAA	Q61K	61	CAA	CAC	Q61H
600	GTG	GAG	V600E	61	CAA	CTA	Q61L	61	CAA	CAT	Q61H
600	GTG	AGG	V600R	61	CAA	CAC	Q61H				
600	GTG	AAG	V600K	61	CAA	CAT	Q61H				
600	GTG	GAT	V600D	146	GCA	ACA	A146T				
601	AAA	GAA	K601E	146	GCA	GTA	A146T				

False negative = sample incorrectly identified as not having any mutations (wild type) listed in Table 2.

### Specificity or Sample-level Negative Agreement

Specificity = # of True negatives / (# of True negatives + # of False positives)

### Mutation-Level Positive Agreement (Sensitivity)

The sensitivity of the KBN-SNPE assay is a measure of its ability to identify samples that contain a mutation listed in Table 2 in *KRAS*, *BRAF,* and *NRAS.* The identification of a mutation present in a highly sensitive assay indicates a high probability of the mutation being present. Precision may also be referred to as the precision of an assay. Sensitivity is defined as follows:

### Sensitivity or Sample Level Positive Agreement

Sensitivity = # of True positives / (# of True positives + # of False negatives)

### Overall Concordance (Accuracy)

The overall accuracy of the KBN*-*SNPE assay to make the same wild type or mutation call as the consensus call is defined as follows:

Overall concordance = (# of True positives + # of True negatives) / (# of True positives + # of False positives + # of False negatives + # True negatives)

## Results

### *KRAS, BRAF NRAS* SNPE Mutation Detection Assay

**Figure 1:**
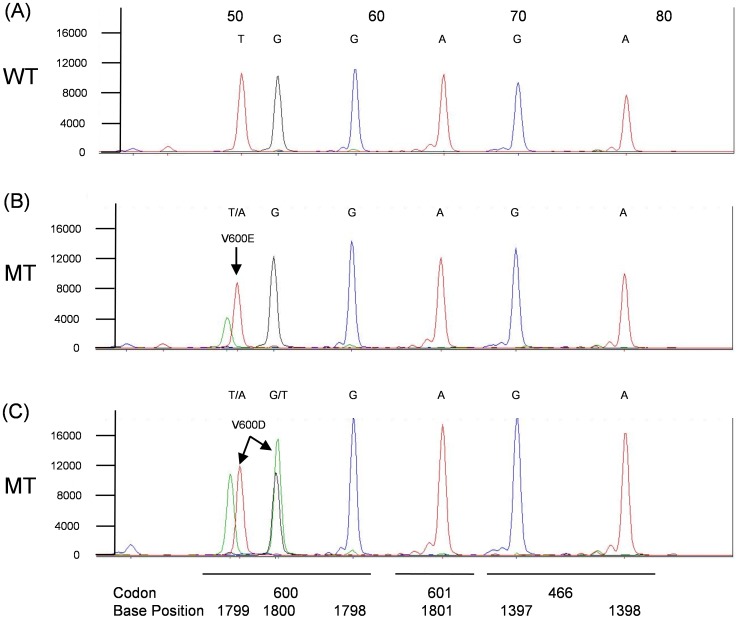
KBN-SNPE results for *BRAF* Probe Pool 7. Panel A: wild type control. Panels B–C: Examples of V600E and V600D mutations. Positions of codons and nucleotides are indicated at the bottom of the figure. Nucleotide label for each peak have been converted to the sense strand.

**Figure 2:**
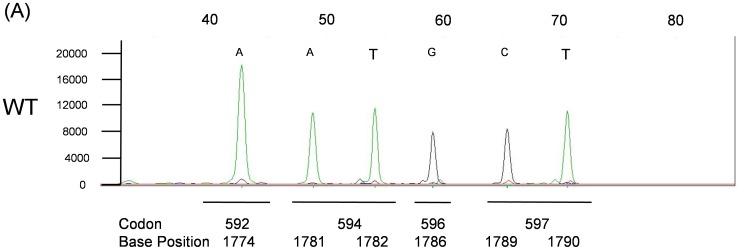
KBN-SNPE results for *BRAF* Probe Pool 6. Panel A: wild type control. Positions of codons and nucleotides are indicated at the bottom of the figure. Nucleotide label for each peak have been converted to the sense strand.

**Figure 3:**
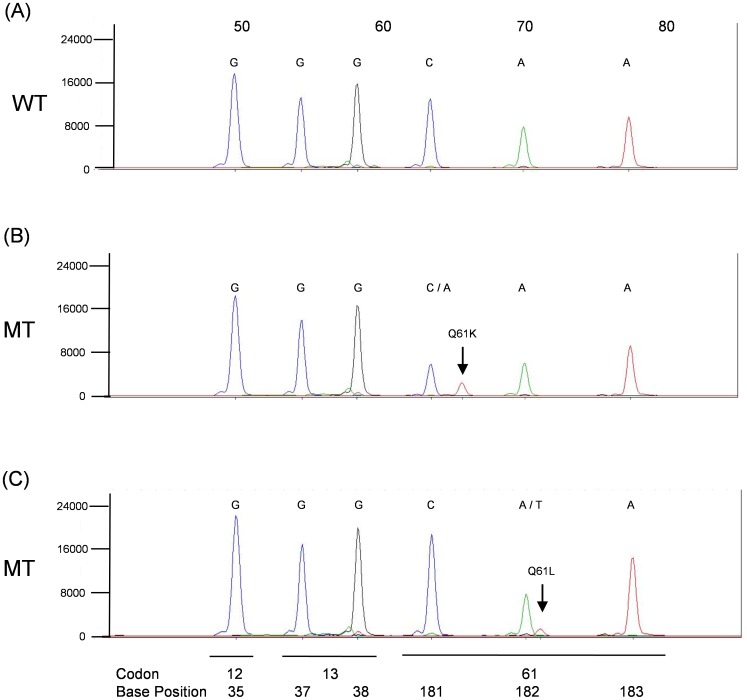
KBN-SNPE results for *NRAS* Probe Pool. Panel A: wild type control. Panels B–C: Examples of Q61K and Q61L mutations. Positions of codons and nucleotides are indicated at the bottom of the figure. Nucleotide label for each peak have been converted to the sense strand.

**Figure 4:**
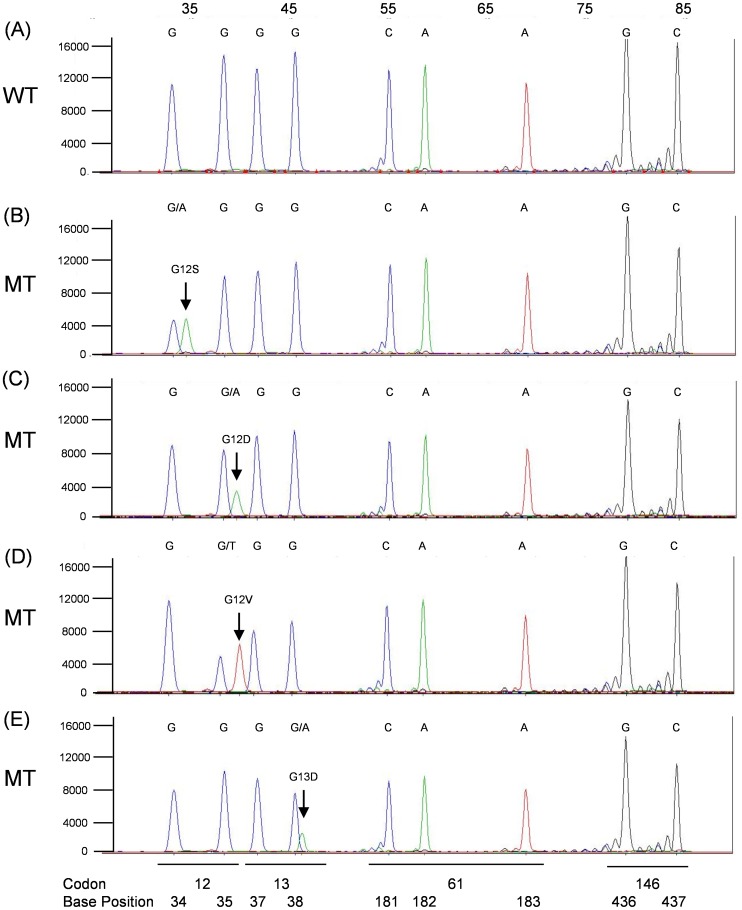
KBN-SNPE results for *KRAS* Probe Pool. Panel A: wild type control. Panels B–E: Examples of G12S, G12D, G12V and G13D mutations. Positions of codons and nucleotides are indicated at the bottom of the figure. Nucleotide label for each peak have been converted to the sense strand.

The *KRAS*, *BRAF*, *NRAS* SNPE (KBN-SNPE) mutation detection assay begins with the PCR amplification of regions of the *KRAS*, *BRAF* and *NRAS* genes from gDNA isolated from one or two 5 µm µFFPE slide. A set of probes is hybridized to the amplicons, followed by a single base extension reaction of the probes and capillary electrophoresis separation and automated base call by the GeneMapper software based on size and incorporated fluorescent label. Examples of a KBN-SNPE assay output are shown in Figures 1, 2, 3, 4.

The base called for each location interrogated is given below each electropherogram and indicated if the nucleotide occupying that position is wild type or mutant. Base calling is determined by the migration of the probe together with the color coding for the nucleotide added during the primer extension reaction. Owing to the slight difference in migration rate, resulting from the addition of the fluorescently tagged nucleotide of each probe, it is possible to identify which of four possible nucleotides was added to the probe.

This KBN-SNPE assay was designed to identify the mutations in *KRAS*, *BRAF* and *NRAS* listed in Table 2 in support of patient stratification in clinical trials. The KBN-SNPE assay was designed and validated to perform well with FFPE samples, which generally yield low quality genomic DNA. To work with such samples we found it necessary to design PCR primers that amplify short stretches (100–150 bp) of DNA (Figure S2) and to carefully balance to primer concentrations (Figure S3) so that all regions were equally amplified.

The KBN-SNPE assay simultaneously interrogates nine and six nucleotide positions within *KRAS* and *NRAS*, respectively, and twelve within *BRAF* (Table 2 and Figure S1). *KRAS* exons 2, 3 and 4, *NRAS* exons 2 and 3 and *BRAF* exons 11 and 15 were amplified in three separate PCR reactions (Table S1). These PCR products served as templates for the SNPE reaction used to identify possible mutations. Due to the number of SNPE probes used for *BRAF* (Table S2) and sequence similarity between *KRAS* and *NRAS*, it was necessary to perform the primer extension portion of the assay in four separate reactions (Table S2). An additional challenge in the design of this assay was the requirement for the detection of the *BRAF* V600D (GTG>GAT) mutation in which two adjacent nucleotides are altered. This was accomplished by designing probes to the appropriate regions of the complementary DNA strands (Table S2, Figure S1). In aggregate we could detect 35 specific and 81 potential mutations (counting all possible A/T/G/C changes at each nucleotide location excluding WT) at 27 nucleotide positions in three genes dispersed over seven exons.

### Analytical Accuracy

To establish the analytical accuracy of the assay three sets of samples, cell lines with previously documented mutations in *BRAF* or *KRAS*, synthetic oligonucleotides containing all mutations detected by the KBN-SNPE assay and a set of 60 FFPE tumor samples were analyzed by both the KBN-SNPE assay and Sanger sequencing.

### The KBN-SNPE Assay Detects Known Mutations in Cell Lines

A number of well characterized cell lines with previously defined mutations in *KRAS, BRAF,* and *NRAS* are commercially available. Such samples mimic tumor samples but have the advantage that gDNA isolated is free from contamination by gDNA from “normal adjacent” tissue, as would be the case when isolating gDNA from FFPE tumor samples. As such, gDNA isolated from cell lines that are heterozygous for particular mutations represent a pool of gDNA with a “known” percentage of mutant DNA in a wild type background and represent an ideal source of DNA for determining the limits of detection of an assay (see LOD section below).

Genomic DNA was isolated from 11 cell lines previously identified, by Sanger sequencing, as containing mutations in *KRAS*, *BRAF*, or *NRAS.* Of the eleven cell lines examined 10 produced the expected result in the KBN-SNPE assay (Table 3). Genomic DNA from the single exception (GAK) was subjected to re-sequencing by Sanger sequencing and was determined to be wild type for *BRAF*, *KRAS* and *NRAS*, confirming the SNPE result. It is possible that the *NRAS* mutation was lost (or its mutation frequency dropped below detection limit) during passage of this cell line.

**Table 3:** KBN-SNPE results using gDNA isolated from cell lines with previously identified mutations in *KRAS, BRAF* or *NRAS.*

Cell Line DNA	Cancer Type	Expected Mutation	*KBN*-SNPE
A375	Melanoma	*BRAF*, V600E	*BRAF*, V600E
ES-2	Ovarian	*BRAF*, V600E	*BRAF*, V600E
WM-266-4	Melanoma	*BRAF*, V600D	*BRAF*, V600D
NCI-H358	Lung	*KRAS*, G12C	*KRAS*, G12C
HCT-8	Colon	*KRAS*, G13D	*KRAS*, G13D
SW 527	Breast	*KRAS*, G12V	*KRAS*, G12V
NCI-H2122	Lung	*KRAS*, G12C	*KRAS*, G12C
Panc-1	Pancreatic	*KRAS*, G12D	*KRAS*, G12D
GAK	Melanoma	*NRAS*, Q61L	WT*
HS-294T	Melanoma	WT	WT
ZR-75-1	Breast	WT	WT

* Wild type status confirmed by re-sequencing of sample used in the KBN-SNPE assay.

### The KBN-SNPE Assay Detects Known Mutations in Oligonucleotides

To further establish that the KBN-SNPE assay is able to detect all mutations listed in Table 2, a series of synthetic oligonucleotides (Table S3) was designed to demonstrate the KBN-SNPE assay’s specificity and ability to detect all listed mutations. These synthetic oligonucleotides replaced the PCR product produced from genomic DNA in the KBN-SNPE assay. Otherwise the KBN-SNPE assay was performed the same as if genomic DNA was used. All appropriate wild type sequences were included as well. The KBN-SNPE assay accurately identified all wild type and mutations at each position interrogated (Table 4).

**Table 4:** Performance of the KBN-SNPE assay with synthetic oligonucleotides.

BRAF			KRAS			NRAS		
Oligo-nucleotide	Expected	Observed	Oligo-nucleotide	Expected	Observed	Oligo-nucleotide	Expected	Observed
B-G466G-S	Wild Type	Wild Type	K-G12G-AS	Wild Type	Wild Type	N-G12G-S	Wild Type	Wild Type
B-G466G-AS	Wild Type	Wild Type	K-G12S-AS	Mutant	Mutant	N-G12G-AS	Mutant	Mutant
B-G466E-AS	Mutant	Mutant	K-G12C-AS	Mutant	Mutant	N-G12D-S	Mutant	Mutant
B-G466V-S	Mutant	Mutant	K-G12D-AS	Mutant	Mutant	N-G12V-S	Wild Type	Wild Type
B-I592I-AS	Wild Type	Wild Type	K-G12A-AS	Mutant	Mutant	N-G13D-S	Mutant	Mutant
B-I592V-AS	Mutant	Mutant	K-G12V-AS	Mutant	Mutant	N-G13R-AS	Mutant	Mutant
B-D594D-S	Wild Type	Wild Type	K-G13G-AS	Wild Type	Wild Type	N-Q61Q-S	Mutant	Mutant
B-D594D-AS	Wild Type	Wild Type	K-G13R-AS	Mutant	Mutant	N-Q61Q-AS	Wild Type	Wild Type
B-D594V-AS	Mutant	Mutant	K-G13D-AS	Mutant	Mutant	N-Q61K-S	Mutant	Mutant
B-D594E-S	Mutant	Mutant	K-Q61Q-S	Wild Type	Wild Type	N-Q61R-AS	Mutant	Mutant
B-D594E-S	Mutant	Mutant	K-Q61Q-AS	Mutant	Mutant	N-Q61L-AS	Mutant	Mutant
B-G596G-S	Wild Type	Wild Type	K-Q61K-S	Mutant	Mutant	N-Q61H-S	Mutant	Mutant
B-G596G-AS	Wild Type	Wild Type	K-Q61L-AS	Mutant	Mutant	N-Q61H-S	Mutant	Mutant
B-G596R-S	Mutant	Mutant	K-Q61H-S	Mutant	Mutant			
B-L597S-S	Mutant	Mutant	K-Q61H-S	Mutant	Mutant			
B-L597S-AS	Mutant	Mutant	K-A146A-S	Wild Type	Wild Type			
B-V600V-S	Wild Type	Wild Type	K-A146A-AS	Mutant	Mutant			
B-V600V-AS	Wild Type	Wild Type	K-A146T-AS	Mutant	Mutant			
B-V600E-AS	Mutant	Mutant	K-A146T-S	Mutant	Mutant			
B-V600R-AS	Mutant	Mutant	K-A146V-S	Mutant	Mutant			
B-V600K-AS	Mutant	Mutant	K-A146V-AS	Mutant	Mutant			
B-V600D-S	Mutant	Mutant						
B-V600D-AS	Mutant	Mutant						
B-K601E-S	Mutant	Mutant						

### The KBN-SNPE Assay Detects Mutations in FFPE Tumor Samples

Genomic DNA was isolated from sixty FFPE samples, ten samples each of colorectal, ovarian, melanoma, lung (non-small cell carcinomas), pancreatic, and thyroid cancers. Each sample was subjected to mutation analysis by the KBN*-*SNPE assay and Sanger sequencing as described in the Methods section. The results are listed in Table 5 and in Table 6.

**Table 5:** Analytical Accuracy: Mutation Analysis of Genomic DNA by KBN-SNPE and Sanger Sequencing.

	*KRAS*			*NRAS*			*BRAF*		
Sample ID	KBN-SNPE	Sequencing	NGS	KBN-SNPE	Sequencing	NGS	KBN-SNPE	Sequencing	NGS
Colorectal-1	WT	WT		Q61L	Q61L		WT	WT	
Colorectal-2	WT	WT		WT	WT		WT	WT	
Colorectal-3	WT	WT		WT	WT		WT	WT	
Colorectal-4	G13D	G13D		WT	WT		WT	WT	
Colorectal-5	WT	WT		WT	WT		WT	WT	
Colorectal-6	G12S	G12N	G12S	WT	WT	WT	WT	WT	WT
Colorectal-7	G12V	G12V		WT	WT		WT	WT	
Colorectal-8	WT	NC	WT	WT	WT	WT	NC	WT	WT
Colorectal-9	WT	WT		WT	WT		WT	WT	
Colorectal-10	WT	WT		WT	WT		V600E	V600E	
Ovarian-1	WT	WT		WT	WT		WT	WT	
Ovarian-2	WT	WT		WT	WT		WT	WT	
Ovarian-3	WT	WT		WT	WT		WT	WT	
Ovarian-4	WT	WT		WT	WT		WT	WT	
Ovarian-5**	WT	NC	ND	NC	NC	ND	WT	G466R*	ND
Ovarian-6	WT	WT		WT	WT		WT	WT	
Ovarian-7	WT	WT		WT	WT		WT	WT	
Ovarian-8	WT	WT	WT	WT	WT	WT	WT	WT	WT
Ovarian-9	WT	WT		WT	WT		WT	WT	
Ovarian-10	WT	WT		WT	WT		WT	WT	
Thyroid-1	WT	WT		WT	WT		V600E	V600E	
Thyroid-2	WT	WT		WT	WT		V600E	V600E	
Thyroid-3	WT	WT		WT	WT		V600E	V600E	
Thyroid-4	WT	Q61Stop*, A146T	WT	WT	WT	WT	V600E	V600E	V600E
Thyroid-5	WT	WT		WT	WT		V600E	V600E	
Thyroid-6	WT	WT		WT	WT		V600E	V600E	
Thyroid-7	WT	WT	WT	WT	G13D	WT	V600E	V600E	V600E
Thyroid-8	A146T*	WT	A146T	WT	WT	WT	V600E	V600E	WT
Thyroid-9	WT	WT		WT	WT		V600E	V600E	
Thyroid-10	WT	WT		WT	WT		WT	WT	
Melanoma-1	WT	WT		WT	WT		WT	WT	
Melanoma-2**	WT	NC	ND	WT	WT	ND	WT	WT	ND
Melanoma-3	WT	WT		Q61K	Q61K		WT	WT	
Melanoma-4**	WT	NC	ND	WT	WT	ND	WT	WT	ND
Melanoma-5**	WT	NC	ND	WT	NC	ND	WT	NC	ND
Melanoma-6**	WT	WT	ND	WT	G13D	ND	V600E	WT	ND
Melanoma-7**	WT	NC	ND	WT	WT	ND	WT	WT	ND
Melanoma-8	WT	WT	WT	WT	WT	WT	WT	WT	WT
Melanoma-9**	WT	NC	ND	WT	WT	ND	NC	WT	ND
Melanoma-10	WT	WT		WT	WT		WT	WT	
Pancreatic-1	WT	WT	WT	WT	WT	WT	WT	NC	WT
Pancreatic-2	WT	G13D	WT	WT	WT	WT	WT	WT	WT
Pancreatic-3	G12D	G12D		WT	WT		WT	WT	
Pancreatic-4	G12D	NC	G12D	WT	WT	WT	WT	WT	WT
Pancreatic-5	G12D	WT	WT	WT	WT	WT	WT	NC	WT
Pancreatic-6	G12D	WT	WT	WT	WT	WT	WT	WT	WT
Pancreatic-7	G12C	NC	WT	WT	WT	WT	WT	WT	WT
Pancreatic-8	WT	WT		WT	WT		WT	WT	
Pancreatic-9	WT	WT		WT	WT		WT	WT	
Pancreatic-10	WT	WT		WT	WT		WT	WT	
Lung-1	WT	WT		WT	WT		WT	WT	
Lung-2	WT	NC	WT	WT	G13D	WT	WT	WT	WT
Lung-3	WT	WT		WT	WT		WT	WT	
Lung-4	WT	WT		WT	WT		WT	WT	
Lung-5	WT	WT		WT	WT		WT	WT	
Lung-6	WT	WT		WT	WT		WT	WT	
Lung-7	G12D	G12D		WT	WT		WT	WT	
Lung-8	WT	WT		WT	WT		WT	WT	
Lung-9	WT	WT		WT	WT		WT	WT	
Lung-10	WT	WT		WT	WT		WT	WT	

* These results were only observed once and have not been reproduced.

** Insufficient material remaining for NGS analysis (ND).

**Table 6:** Percentage of tumors with mutations in *KRAS*, *BRAF* or *NRAS* as reported in the COSMIC Database and this study.

	Cosmic			This Study		
	KRAS	BRAF	NRAS	KRAS	BRAF	NRAS
Colorectal	35	12	2	30	10	10
Melanoma	2	43	13	0	10	10
Ovarian	14	9	1	0	0	0
Pancreatic	58	2	1	20	0	0
Thyroid	2	40	0	10	90	0
Lung	16	2	1	10	0	0

In general we observed the expected number and type of mutations based on the COSMIC database (Table 6). The exceptions seem to be under reporting of *BRAF* and *KRAS* mutations in melanomas and pancreatic cancers respectively (10% and 20% in this study vs. 43% and 58% respectively reported in COSMIC). *BRAF* mutations in thyroid cancers appear to be over reported (90% in this study vs. 40% in COSMIC). It should be noted that the rates of mutations reported in COSMIC also differs from some reported rate cited on the literature [3]–[12]. Mutation rates reported in the COSMIC database include all mutations reported for these genes, while rates reported in the literature, as well as this study, focus on a more restricted set of mutations. The small sample size used in this study may also contribute to any discrepancy observed.

Discordant results between KBN-SNPE and Sanger results were observed in 18 out of 60 samples. To resolve these discordant results, samples were analyzed by Ion PGM to produce a consensus result (see Methods for details). There was insufficient material for NGS analysis for seven (one ovarian and six melanoma cancers) of the eighteen discordant samples. NGS results for seven of the eleven discordant samples agreed with the KBN-SNPE assay results. The remaining four samples agreed with Sanger sequencing results.

### Intra-Run Variation (Repeatability)

To assess intra-run precision, eight samples from the Accuracy study were run in replicas of eight in a single batch using the standard KBN-SNPE multiplexing assay (see Methods). The results were recorded and compared to the consensus call (see Methods). Precision was 100% for six of the eight samples; one discordant result was observed for each of the two ovarian samples. *KRAS* exon 2 also failed to amplify in one replica of Ovarian-1 as well (Table 7).

**Table 7:** Intra-Run Variation (Repeatability).

	Colorectal-1			Colorectal-7			Thyroid-2			Thyroid-8		
	*KRAS*	*NRAS*	*BRAF*	*KRAS*	*NRAS*	*BRAF*	*KRAS*	*NRAS*	*BRAF*	*KRAS*	*NRAS*	*BRAF*
Truth	WT	Q61L	WT	G12V	WT	WT	WT	WT	V600E	WT	WT	V600E
1	WT	Q61L	WT	G12V	WT	WT	WT	WT	V600E	WT	WT	V600E
2	WT	Q61L	WT	G12V	WT	WT	WT	WT	V600E	WT	WT	V600E
3	WT	Q61L	WT	G12V	WT	WT	WT	WT	V600E	WT	WT	V600E
4	WT	Q61L	WT	G12V	WT	WT	WT	WT	V600E	WT	WT	V600E
5	WT	Q61L	WT	G12V	WT	WT	WT	WT	V600E	WT	WT	V600E
6	WT	Q61L	WT	G12V	WT	WT	WT	WT	V600E	WT	WT	V600E
7	WT	Q61L	WT	G12V	WT	WT	WT	WT	V600E	WT	WT	V600E
8	WT	Q61L	WT	G12V	WT	WT	WT	WT	V600E	WT	WT	V600E

	Lung-7			Pancreactic-3			Ovarian-1			Ovarian-8		
	*KRAS*	*NRAS*	*BRAF*	*KRAS*	*NRAS*	*BRAF*	*KRAS*	*NRAS*	*BRAF*	*KRAS*	*NRAS*	*BRAF*
Truth	G12D	WT	WT	G12D	WT	WT	WT	WT	WT	WT	WT	WT
1	G12D	WT	WT	G12D	WT	WT	WT	WT	WT	WT	WT	WT
2	G12D	WT	WT	G12D	WT	WT	WT	WT	WT	WT	WT	WT
3	G12D	WT	WT	G12D	WT	WT	WT	WT	WT	WT	G13D	WT
4	G12D	WT	WT	G12D	WT	WT	WT	WT	WT	WT	WT	WT
5	G12D	WT	WT	G12D	WT	WT	No Exon 2	WT	WT	WT	WT	WT
6	G12D	WT	WT	G12D	WT	WT	WT	G12D	WT	WT	WT	WT
7	G12D	WT	WT	G12D	WT	WT	WT	WT	WT	WT	WT	WT
8	G12D	WT	WT	G12D	WT	WT	WT	WT	WT	WT	WT	WT

### Inter-Run Variation (Reproducibility)

To assess the inter-run precision, i.e., how the assay performs when the samples are assayed over a period of time, eight genomic DNA samples were assayed 8 times in 8 batches over a period of four days (Table 8). 100% precision was observed for 5 of the 8 samples. A thyroid sample and a CRC sample each had one false positive in NRAS, whereas one melanoma sample had two false negatives in BRAF and a false positive in KRAS.

**Table 8:** Inter-Run Variation (Reproducibility).

Colorectal-1			Colorectal-10			Thyroid-1			Thyroid-8		
*KRAS*	*NRAS*	*BRAF*	*KRAS*	*NRAS*	*BRAF*	*KRAS*	*NRAS*	*BRAF*	*KRAS*	*NRAS*	*BRAF*
WT	Q61L	WT	WT	WT	V600E	WT	WT	V600E	WT	WT	V600E
WT	Q61L	WT	WT	WT	V600E	WT	WT	V600E	WT	WT	V600E
WT	Q61L	WT	WT	WT	V600E	WT	WT	V600E	WT	WT	V600E
WT	Q61L	WT	WT	WT	V600E	WT	WT	V600E	WT	WT	V600E
WT	Q61L	WT	WT	WT	V600E	no Ex2	WT	V600E	WT	WT	V600E
WT	Q61L	WT	WT	WT	V600E	no Ex2	WT	V600E	WT	WT	V600E
WT	Q61L	WT	WT	G13D	V600E	WT	G13D	V600E	WT	WT	V600E
WT	Q61L	WT	WT	WT	V600E	WT	WT	V600E	WT	WT	V600E
WT	Q61L	WT	WT	WT	V600E	WT	WT	V600E	WT	WT	V600E

Ovarian-1			Ovarian-8			Lung-7			Melanoma-6		
KRAS	NRAS	BRAF	KRAS	NRAS	BRAF	KRAS	NRAS	BRAF	KRAS	NRAS	BRAF
WT	WT	WT	WT	WT	WT	G12D	WT	WT	WT	WT	V600E
WT	WT	WT	WT	WT	WT	G12D	WT	WT	WT	WT	V600E
WT	WT	WT	WT	WT	WT	G12D	WT	WT	WT	WT	V600E
WT	WT	WT	WT	WT	WT	G12D	WT	WT	WT	WT	V600E
WT	WT	WT	WT	WT	WT	G12D	WT	WT	WT	WT	V600E
WT	WT	WT	WT	WT	WT	G12D	WT	WT	WT	WT	V600E
WT	WT	WT	WT	WT	WT	G12D	WT	WT	WT	WT	WT
WT	WT	WT	WT	WT	WT	G12D	WT	WT	WT	WT	WT
WT	WT	WT	WT	WT	WT	G12D	WT	WT	G12D	WT	V600E

### Limit of Detection (Analytical Sensitivity)

For a qualitative assay, analytical sensitivity (limit of detection, LOD) is the lowest percentage of mutant gDNA mixed with wild type gDNA where a known mutation can be detected (CLSI guideline EP17, Protocols for Determination of Limits of Detection and Limits of Quantitation). Aliquots of gDNA from mutant cell lines (two *BRAF* and three *KRAS*; Table 9) were mixed with wild type gDNA at ratios of 25%, 15%, 10%, 5% and 1% of mutant to wild type gDNA and then analyzed by the KBN-SNPE assay. No *NRAS* mutant cell lines were available from ATCC so the LOD for *NRAS* mutations could not be performed. The LOD was dependent on the actual mutation or cell line used for the experiment and varied from 1.6% to 12%. One KRAS mutation was detected at 1.6% mutant to wild type DNA; two other mutations were detected at 6% mutant DNA and all mutations were detected at 12% mutant DNA.

**Table 9:** Lowest percentage of mutant DNA in wild type background and still detect mutation.

	*BRAF*		*KRAS*		
Cell Line	ES2	A375	HCT8	Panc1	SW527
Mutation	V600E	V600E	G13D	G12D	G12V
% Mut DNA					
25	Yes	Yes	Yes	Yes	Yes
12.5	Yes	Yes	Yes	Yes	Yes
6.25	Yes	No	No	Yes	No
3.125	No	No	No	Yes	No
1.56	No	No	No	Yes	No
0.78	No	No	No	No	No

Interpretation of these results is confounded by a number of issues. First, it is difficult to accurately determine the starting percentage of mutant DNA present in a given sample. For these calculations we assumed each cell line was heterozygous for the mutation. Second, the differences in extinction coefficients for the fluorescent dyes used to identify the various bases incorporated into the KBN-SNPE primer makes it difficult to accurately assess the relative ratios of one nucleotide verses another. A conservative estimate of the LOD is between 6 and 12% mutant DNA. No matrix effect was detected for this study (data not shown).

### Sample Analysis in Triplicate Improves Assay Performance

In the experiments described above it was noted that miscalls could occur at a rate of 2–3 %. For clinical applications, it would be desirable to reduce this error rate as much as possible. We speculated that we could reduce the error rate of the assay by assaying samples in triplicate and applying a ‘majority rules’ approach (see Methods). Rules for calling KBN-SNPE results are given in Table 1.

The results, by gene, of 14 samples which were assayed in triplicate on three independent occasions are listed in Table 10. There were no missed calls in the *BRAF* and *NRAS* portions of the assay. There were three indeterminate calls (one melanoma-2 and two with thyroid-1) for the *KRAS* portion of the assay. Under the redo rules for this assay the melanoma-2 would have been repeated and its mutation status reported as “wild type”. Thyorid-1 would have been repeated and its mutation status reported as “indeterminate”. Therefore, when samples were assayed in triplicate there was 100% (42/42) concordance between the KBN-SNPE result and the consensus result (defined by agreement with Sanger or NGS). Samples assayed in triplicate where two or more calls where indeterminate (ND) were excluded from calculations for concordance.

**Table 10:** Accuracy rates for *BRAF/KRAS/NRAS* when samples were assayed in triplicate.

		*BRAF*									
		Experiment 1			Experiment 2			Experiment 3			
Sample	Consensus	Rep 1	Rep 2	Rep 3	Rep 1	Rep 2	Rep 3	Rep 1	Rep 2	Rep 3	Concordance
Colorectal-1	WT	WT	WT	WT	WT	WT	WT	WT	WT	WT	3/3
Colorectal-4	WT	WT	WT	WT	WT	WT	WT	WT	WT	WT	3/3
Colorectal-7	WT	WT	WT	WT	WT	WT	WT	WT	WT	WT	3/3
Colorectal-10	V600E	V600E	V600E	V600E	V600E	V600E	V600E	V600E	V600E	V600E	3/3
Melanoma-2	WT	WT	WT	WT	WT	WT	WT	WT	WT	WT	3/3
Melanoma-3	WT	WT	WT	WT	WT	WT	WT	WT	WT	WT	3/3
Ovarian-1	WT	WT	WT	WT	WT	WT	WT	WT	WT	WT	3/3
Ovarian-8	WT	WT	WT	WT	WT	WT	WT	WT	WT	WT	3/3
Pancreatic-3	WT	WT	WT	WT	WT	WT	WT	WT	WT	WT	3/3
Pancreatic-8	WT	WT	WT	WT	WT	WT	WT	WT	WT	WT	3/3
Thyroid-1	V600E	V600E	V600E	V600E	V600E	V600E	V600E	V600E	V600E	V600E	3/3
Thyroid-8	V600E	V600E	V600E	V600E	V600E	V600E	V600E	V600E	V600E	V600E	3/3
Lung-5	WT	WT	WT	WT	WT	WT	WT	WT	WT	WT	3/3
Lung-7	WT	WT	WT	WT	WT	WT	WT	WT	WT	WT	3/3

		*KRAS*														
		Experiment 1					Experiment 2						Experiment 3			
Sample	Consensus	Rep 1	Rep 2		Rep 3		Rep 1		Rep 2		Rep 3		Rep 1	Rep 2	Rep 3	Concordance
Colorectal-1	WT	WT	WT		WT		WT		WT		WT		ND*	WT	WT	3/3
Colorectal-4	G13D	G13D	G13D		G13D		G13D		G13D		G13D		G13D	G13D	G13D	3/3
Colorectal-7	G12V	G12V	G12V		G12V		G12V		G12V		G12V		G12V	G12V	G12V	3/3
Colorectal-10	WT	WT	WT		WT		WT		WT		WT		WT	WT	WT	3/3
Melanoma-2	WT	WT	ND*		ND		WT		WT		ND		WT	WT	ND	2/2
Melanoma-3	WT	WT	WT		WT		WT		WT		WT		WT	WT	WT	3/3
Ovarian-1	WT	WT	WT		WT		WT		WT		WT		WT	WT	WT	3/3
Ovarian-8	WT	WT	WT		WT		WT		WT		WT		WT	G12D	WT	3/3
Pancreatic-3	G12D	G12D	G12D		G12D		G12D		G12D		G12D		G12D	G12D	G12D	3/3
Pancreatic-8	WT	WT	WT		WT		WT		WT		ND		WT	WT	ND	3/3
Thyroid-1	WT	ND	A146T		WT		ND		ND		WT		ND	WT	WT	1/2
Thyroid-8	WT	WT	WT		A146V		WT		WT		WT		WT	WT	WT	3/3
Lung-5	WT	WT	WT		WT		WT		WT		WT		WT	WT	WT	3/3
Lung-7	G12D	G12D	G12D		G12D		G12D		G12D		G12D		G12D	G12D	G12D	3/3
		***NRAS***														
		**Experiment 1**						**Experiment 2**					**Experiment 3**			
**Sample**	**Consensus**	**Rep 1**		**Rep 2**		**Rep 3**		**Rep 1**		**Rep 2**		**Rep 3**	**Rep 1**	**Rep 2**	**Rep 3**	**Concordance**
Colorectal-1	QA1L	Q61L		Q61L		Q61L		Q61L		Q61L		Q61L	Q61L	Q61L	Q61L	3/3
Colorectal-4	WT	WT		WT		WT		WT		WT		WT	WT	WT	WT	3/3
Colorectal-7	WT	WT		WT		WT		WT		WT		WT	WT	WT	WT	3/3
Colorectal-10	WT	WT		WT		WT		WT		WT		WT	G12D	WT	WT	3/3
Melanoma-2	WT	WT		WT		WT		WT		WT		WT	WT	WT	WT	3/3
Melanoma-3	Q61K	Q61K		Q61K		Q61K		Q61K		Q61K		Q61K	Q61K	Q61K	Q61K	3/3
Ovarian-1	WT	WT		WT		WT		WT		WT		WT	WT	Q61L	WT	3/3
Ovarian-8	WT	WT		WT		WT		WT		WT		WT	WT	WT	WT	3/3
Pancreatic-3	WT	WT		WT		WT		WT		WT		WT	WT	WT	WT	3/3
Pancreatic-8	WT	WT		WT		WT		WT		WT		WT	WT	WT	WT	3/3
Thyroid-1	WT	WT		WT		WT		WT		WT		WT	G12D, G13V	WT	WT	3/3
Thyroid-8	WT	WT		WT		WT		WT		WT		WT	WT	WT	WT	3/3
Lung-5	WT	WT		WT		WT		WT		WT		WT	WT	WT	WT	3/3
Lung-7	WT	WT		WT		WT		WT		WT		WT	WT	WT	WT	3/3

* ND-No data due to one or more exons missing

 One envisioned application of the KBN-SNPE assay is for its use as a clinical trial assay to select patients whose tumor displays an activated MAPK pathway which may make it more susceptible to MAPK inhibitors. Binary classification is the process of classifying objects within a group (cancer patients) into two subsets, cancer patients with mutations in *KRAS*, *BRAF* or *NRAS*, and cancer patients without mutations in these genes. How well an assay performs in this binary classification of patients is measured by its concordance, or accuracy, and its specificity and sensitivity. For the KBN-SNPE assay, sensitivity is the ability of the assay to correctly identify samples containing a mutation listed in Table 2. An additional measure of assay performance is the assay specificity, or its ability to correctly identify samples that lack any of the *KRAS*, *BRAF* and *NRAS* mutations listed in Table 2. An ideal assay would be 100% for both specificity and sensitivity. Table 11 gives the performance characteristics for the KBN-SNPE assay at the gene level. Intra-run precision for the *BRAF*, *KRAS* and *NRAS* genes was 100% (64/64), 100% (63/63) and 97% (62/64), respectively. Inter-run precision for the *BRAF*, *KRAS* and *NRAS* genes was 97% (62/64), 98% (61/62) and 97% (62/64), respectively. Table 12 gives the performance characteristics at the sample or patient level. For patient stratification, in a clinical trial, patient-level analysis is more appropriate than the gene-level analysis described above.

**Table 11:** Performance characteristics for the KBN-SNPE at the gene level.

Samples Analyzed	Specificity or Sample-Level Negative Agreement			Sensitivity or Mutation-Level Positive Agreement			Overall Concordance (Accuracy)		
	KRAS	NRAS	BRAF	KRAS	NRAS	BRAF	KRAS	NRAS	BRAF
FFPE Tumor samples, Screen	94% (44/47)	100% (55/55)	100% (46/46)	100% (7/7)	100% (2/2)	100% (10/10)	94% (51/54)	100% (55/55)	100% (51/51)
FFPE Assayed in Triplicate	100% (27/27)	100% (36/36)	100% (33/33)	100% (12/12)	100% (6/6)	100% (9/9)	100% (39/39)	100% (48/48)	100% (42/42)
Intra-Run Variation	100% (39/39)	96% (54/56)	100% (48/48)	100% (24/24)	80% (8/10)	100% (16/16)	100% (63/63)	97% (62/64)	100% (64/64)
Inter-Run Variation	98% (53/54)	96% (54/56)	100% (32/32)	100% (8/8)	100% (8/8)	94% (30/32)	98% (61/62)	97% (62/64)	97% (62/64)
Cell line DNA	100% (6/6)	100% (11/11)	100% (8/8)	100% (5/5)	100% (0/0)	100% (3/3)	100% (11/11)	100% (11/11)	100% (11/11)

**Table 12:** Performance characteristics for the KBN-SNPE at the sample, or patient, level.

Samples Analyzed	Specificity or Sample-level Negative Agreement	Sensitivity or Mutation-levelPositive Agreement	Overall Concordance(Accuracy)
FFPE Tumor samples, Screen	91% (32/35)	100% (18/18)	94% (50/53)
FFPE Tumor samples assayed in Triplicate	100% (13/13)	100% (25/25)	100% (38/38)
Intra-Run Variation	87% (13/15)	100% (48/48)	97% (61/63)
Inter-Run Variation	100% (16/16)	96% (44/46)	97% (60/62)
Cell line DNA	100% (3/3)	100% (8/8)	100% (11/11)

## Discussion

In this paper, we described the development and analytical validation of a custom designed, multiplexed mutation assay to detect 35 mutations of interest in *KRAS*, *BRAF*, and *NRAS* genes in FFPE tumor tissue for patient stratification in clinical trials. We evaluated the assay in 6 different tumor types procured from commercial sources for the assay validation and compared our KBN-SNPE (*KRAS*, *BRAF*, *NRAS* Single Nucleotide Primer Extension) mutation assay results to Sanger sequencing and NGS. Most discordant results were resolved in favor of the SNPE assay as summarized in Table 5. Using available cultured cell lines and synthetic oligonucleotide templates with mutations of interest, we were able to validate the specificity of our primer/probe designs in these custom designed multiplexing SNPE assays. At the sample level this assay has a concordance of 94% when samples were assayed as a single point and 100% when samples were assayed in triplicate and a sensitivity (precision) of nearly 100% (Table 12).The limit of detection varied from 2–12%, depending on the mutation.

### Assay Design and Optimization Considerations

The assay includes multiplexing of both the PCR template amplifications and the SNPE reactions. The competition among the PCR primers is not only against the gDNA templates but also against each other. These primers also compete against nucleotides and DNA polymerase both in the kinetic and thermodynamic manner depending on their size, sequence, and melting temperature. Figure S2 shows the example of multiplex PCR amplification of *KRAS* exons 2 and 3, as equal concentrations of primers and gDNA template resulting in different degree of amplification of these PCR templates. We found that when more than two PCR primer sets or primers/probes are used, changing one primer concentration will likely result in changes in all PCR products or SNPE products (data not shown). Similar results were observed when more than 10 SNPE primers/probes were used to interrogate large number of mutation hot spots. Strong competition between primer pairs makes adjustment of relative peak heights a challenging task, since changing the concentration of one primer will result in re-equilibration of many other primers/probes.

The amplicon size for the PCR template is very critical to the success of mutation detection with FFPE tissue samples using this platform. Initial design efforts and assay optimization were conducted using normal human blood gDNA samples. While data generated using longer PCR template containing mutation hotspots were very successful, when FFPE tumor tissue samples were used, amplification of target regions was inconsistent (Figure S3), presumably because the DNA from FFPE tissue samples was highly fragmented and or degraded. Compromises were made to design shorter amplicons in order to accommodate such low quality samples that are common in FFPE tissues. Other challenges encountered included designing SNPE primers to detect certain double mutations involving two consecutive nucleotide changes, identifying cell lines with specific mutations as control or reference samples, and detecting low frequency mutations using limited amount of FFPE tissue samples. We will further discuss special experimental designs and proposed mitigation plans in this section.

### Designing SNPE Primers to Detect Certain Mutations Involving Two Consecutive Nucleotide Changes

The detection of *BRAF* V600K and V600R mutations presented special challenges in SNPE probe design. In addition to designing oligonucleotide primers/probes from both sense and anti-sense directions, it was necessary to design additional primers/probes in order to cover potential mutant populations as shown in Table 3, BRAF2 pools where 1799(A)/600 probe covers any mutants with A at 1799 nucleotide position to allow detecting mutants with double mutation such as V600R (GTG to AGG) This design is necessary in order to detect the double mutations including a more aggressive form of *BRAF* mutation V600K which has drawn a lot of attention recently for its possible link to the tumor metastasis [25]–[26].

### Oligonucleotides Containing Mutations of Interest as Templates for Assay Validation

Since it is very difficult to obtain cell lines with all of the specific mutations of interest to serve as control samples or for assay validation purposes, we employed a strategy of using oligonucleotide templates containing wild-type and mutant sequences in both sense and anti-sense directions. We used these oligonucleotides to validate our primers/probes for their specificity in detecting each mutation of interest. These oligonucleotides are synthetic single-stranded templates mimicking the PCR products. Therefore, there are no fundamental difference between using these synthetic templates and using the PCR products from cell lines with specific mutations.

### Potential Impact of Limited Quantity and Low Quality of gDNA Isolated from the Clinical Samples

One challenge frequently encountered during the clinical sample testing is that many samples fail to meet minimum sample requirements, either due to the low quality or due to insufficient quantity. Efforts throughout the biomarker community typically focus on acquiring high quality preclinical and clinical bio-samples in order to facilitate biomarker discovery and development. However, from a clinical assay development point of view, this approach may have unintended consequences, since it may lead to assays that are difficult to clinically validate with actual clinical samples. Without solving the sample collection and storage issues at the clinical sites, the use of high quality samples for biomarker discovery and development may make it difficult to reproduce an observed biomarker effect in a clinical setting. Therefore, in biomarker discovery-development stage, pre-clinical samples with quality that mimics those of clinical samples expected from a clinical trial should be evaluated. This could be done by using preclinical samples that went through controlled degradation/fragmentation process and use them for the pre-clinical biomarker validation.

### Key Considerations in Detecting Low Allele Frequency Mutations

Another challenge, closely related to the above quality/quantity issue, is the need to detect low frequency mutations. Using FFPE tissue samples as an example, in order to detect specific mutations intact target gDNA fragments are needed to allow PCR primers to hybridize and initiate PCR amplification. High quality samples with low quantity gDNA or low quality samples with high quantity gDNA may both result in a failure to detect low allele frequency mutants. The important question to ask is how many copies of the gDNA with intact target sequences are available in the initial PCR template amplification reaction. In order to detect 1% of mutant DNA, at least 1000 copies of intact DNA containing the complete target sequence is needed, otherwise, sampling variation will likely result in a detection range of 0% to possibly greater than 5%, depending on the sampling chances. For human genomic DNA, 1000 copies translate into approximately 3 ng of intact DNA. If 30 ng of gDNA isolated from a fragmented FFPE tissue slide contains 10% of intact target DNA fragment, the chance of using an aliquot of this batch of DNA to accurately detect 1% mutant DNA population is very low especially if only one mutation assay is done without replicates.

### Sensitivity of the KBN-SNPE Assay Compared to Therascreen and Cobas Assays

The manufacturers of both the Therascreen *KRAS* and Cobas *KRAS* and *BRAF* assays state that less than 5% mutant DNA can be detected using 100 ng gDNA isolated from FFPE tissue samples with at least 10% tumor cells on the slide. If we assume 10% of gDNA in the FFPE tissue is intact, this would mean that those samples that met minimum requirements on the slide should have 1 ng intact target DNA or 333 DNA copies available for PCR amplification. Therefore, for 333 copies of target DNA fragment, 5% of mutant population equals to ∼16 copies, and hence if such FFPE tissue slides are used to isolate gDNA and aliquot of it used to perform mutation tests, chance of accurately detecting such low percent mutant population is very low, and those aliquots are likely to contain mutant copies as low as 1% and as high as 8–9% based on the normal distribution of random drawing. One way to avoid this issue is to establish some method of quality control (such as determining the number of amplifiable copies of target DNA) to identify samples that meet minimum quality and quantity requirements.

For our custom designed multiplexing mutation assay, the detection sensitivity is similar to those FDA approved KRAS/BRAF assays mentioned above. In our study as shown in Table 10, triplicate samples were run to determine assay accuracy using 15 ng of gDNA as starting material for each replicates. Several samples showed presumably false positive results as two of the replicates showed no mutations were detected while one of the replicates showed mutation detected. Since the KBN-SNPE assay design employed DNA sequencing-styled fragment separation (based on both migration and color labeling differences), false positives or false negatives are extremely rare. Our interpretation for such result is that those samples might have mutation frequencies slightly below detection sensitivity, and therefore the sampling variation caused one of the replicates to have more copies of mutant DNA than the others and therefore mutation was detected. In order to accurately detect mutation frequency for each sample, a true quantitative mutation assay is required. So far most of the existing mutation detection methodologies are considered “semi-quantitative” at their best. With the arrival of NGS technologies, quantitative measurement of mutation frequency might be possible. However, if small number of amplifiable gDNA copies is available as the starting material a triplicate library preparation strategy for NGS might be necessary to assure accurate determination of clinical samples especially in the case of when low level of mutation detection is critical.

### Assay Sensitivity is Crucial to Accurate and Consistent Diagnosis

Since the mutation assay is to be used for patient treatment decision, it raises the following question. Since patients with specific mutant population of 8–9% might respond to the treatment while those with 1% may not, is it acceptable to get false negative results from patient samples with 8–9% mutant DNA and is it appropriate to enroll patients with samples contain only 1% mutant DNA? Certain low percent mutations (<1%) detected in leukemia samples are very significant in terms of impacting the clinical outcome of corresponding patients [27], while in the clinical trial setting with an experimental therapeutic, samples containing 1% mutation might behave more like wild-type tumors [28]. Interestingly, a recent report has shown that a clinical study used Sanger sequencing for patient enrollment decisions enrolled some patients with their mutation status determined as wild-type. After retesting the samples using recently FDA-cleared KRAS/BRAF tests, some samples were found to harbor mutations [29]. Simply by improving detecting sensitivity from 15% to 5%, many inconsistent results will be identified especially for those mutations present at low frequencies. In addition, many mutations previously believed to be rare are now being detected at higher rates likely due to the improvements of detection sensitivity of new qPCR mutation detection assays and NGS assays, which are reported to permit detection of mutant populations at low single digit percentage level [30], [31]. Thus, these ‘rare mutations’ might be more prevalent than what we originally thought. Now that NGS platforms are readily available for both research institutes and the pharmaceutical industry, this trend is almost guaranteed to continue. The question is, how should patients with very low allele frequency mutations (e.g., detected at below 0.1%) be treated? Are their tumors likely to respond as a mutant or wild-type? Therefore, in a clinical trial, a cutoff percentage filter applied to the mutation frequency determined by a sensitive assay might be necessary according to their clinical relevance for each mutation. Nevertheless, in a clinically practical sense, there is a lower limit in terms of the detection of low percentage mutants. Detection will be limited by the amount of DNA extractable from the clinical specimen. In the case of extracting DNA from a single 5 µm FFPE tissue slide, that number could be as low as 10–100 ng. Therefore, based on the above discussed calculations, accurately detecting 0.1% mutant DNA in a wild-type background for the FFPE tissue slide samples is probably close to the practical limit.

### Conclusion

In conclusion, we have developed and analytically validated a custom designed multiplexing mutation assay called KBN-SNPE assay that is suitable for patient selection for clinical trials. The extensive list of mutations (all MAPK activating mutations) justifies the selection of an assay design based on the SNPE multiplexing assay platform. Many of the assay development and validation issues discussed here may be applicable to other efforts to develop and validate clinical mutation detection assays, including NGS-based clinical assays.

## Supporting Information

Figure S1 PCR Primer Locations and Hot Spots for *BRAF*, *NRAS* and *KRAS.* Sequences highlighted in grey denote regions around which PCR primers were designed. Sequences highlighted with underline are hot spot codons around which SNPE probes were designed.
(DOCX)

Figure S2 Effect of PCR Primer Ratios on Amplification of DNA Fragments. The ability to amplify DNA fragments was dependent on the ratio of the PCR primer sets to each other. Increasing the concentration of one set of primers relative to the second set favored to amplification of one product over the other. The above exercise was repeated with the appropriate PCR primers until all PCR products were amplified to similar amounts as judged by gel analysis.
(DOCX)

Figure S3 Failure of large DNA fragments to be amplified from FFPE derived gDNA in PCR. Large fragments of DNA fail to be amplified from FFPE derived gDNA. Replicate samples of FFPE gDNA from four tumor types (Ovarian cancer, lanes 1, 5, 9; Lung cancer, lanes 2, 6 10; colorectal cancer, lanes 3, 7, 11; pancreatic cancer, lanes 4, 8, 12) were subjected to PCR amplification for *KRAS*, *NRAS* and *BRAF*. PCR primers for *KRAS* were designed to amplify fragments of DNA 200–300 bp in size while PCR primers for *NRAS* and *BRAF* were designed to amplify fragments 125–150 bp in size. Three out of four PCR reactions for *KRAS* failed to successfully amplify DNA fragments while all PCR reactions of *NRAS* and *BRAF* were successful.
(DOCX)

Table S1 Sequences and Working Stocks for PCR Primers and SNPE Reaction Probes.
(DOCX)

Table S2 SNPE Probe Sequences and Pools.
(DOCX)

Table S3 Sequence for Synthetic Oligonucleotides.
(DOCX)
