# Supplementary material for: Development and Validation of a Clinical Trial Patient Stratification Assay That Interrogates 27 Mutation Sites in MAPK Pathway Genes
Source: PLoS One. 2013 Aug 21;8(8):e72239. doi: 10.1371/journal.pone.0072239 (PMC3749116; doi:10.1371/journal.pone.0072239)
Supplement: Figure S1 — PCR Primer Locations and Hot Spots for BRAF, NRAS and KRAS. Sequences highlighted in grey denote regions around which PCR primers were designed. Sequences highlighted with underline are hot spot codons around which SNPE probes were designed. (DOCX) [file pone.0072239.s001.docx]

**Figure S1**

PCR Primer Locations and Hot Spots for *BRAF*, *NRAS* and *KRAS*

Sequences highlighted in grey denote regions around which PCR primers were designed. Sequences highlighted with underline are hot spot codons around which SNPE probes were designed.

*BRAF* Exon 11

GTGATGATTGGGAGATTCCTGATGGGCAGATTACAGTGGGACAAAGAATTGGATCTGGATCATTTGGAAC

AGTCTACAAGGGAAAGTGGCATGGTGATGTGGCAGTGAAAATGTTGAATGTGACAGCACCTACACCTCAG

*BRAF* Exon 15

ATGGATTACTTACACGCCAAGTCAATCATCCACAGAGACCTCAAGAGTAATAATATATTTCTTCATGAAG

ACCTCACAGTAAAAATAGGTGATTTTGGTCTAGCTACAGTGAAATCTCGATGGAGTGGGTCCCATCAGTT

TGAACAGTTGTCTGGATCCATTTTGTGGATGGCACCAGAAGTCATCAGAATGCAAGATAAAAATCCATAC

*NRAS* Exon 2

TTACTGGTTTCCAACAGGTTCTTGCTGGTGTGAAATGACTGAGTACAAACTGGTGGTGGTTGGAGCAGGT

GGTGTTGGGAAAAGCGCACTGACAATCCAGCTAATCCAGAACCACTTTGTAGATGAATATGATCCCACCA

TAGAGGTGAGGCCCAGTGGTAGCCCGCTGACCTGATCCTGTCTCTCACTTGTCGGATCATCTTTACCCAT

*NRAS* Exon 3

ATAGATGGTGAAACCTGTTTGTTGGACATACTGGATACAGCTGGACAAGAAGAGTACAGTGCCATGAGAG

ACCAATACATGAGGACAGGCGAAGGCTTCCTCTGTGTATTTGCCATCAATAATAGCAAGTCATTTGCGGA

*KRAS*

*KRAS* Exon 2

TAGTGTATTAACCTTATGTGTGACATGTTCTAATATAGTCACATTTTCATTATTTTTATTATAAGGCCTG

CTGAAAATGACTGAATATAAACTTGTGGTAGTTGGAGCTGGTGGCGTAGGCAAGAGTGCCTTGACGATAC

AGCTAATTCAGAATCATTTTGTGGACGAATATGATCCAACAATAGAGGTAAATCTTGTTTTAATATGCAT

ATTACTGGTGCAGGACCATTCTTTGATACAGATAAAGGTTTCTCTGACCATTTTCATGAGTACTTATTAC

*KRAS* Exon 3

CCTTTTTTGAAGTAAAAGGTGCACTGTAATAATCCAGACTGTGTTTCTCCCTTCTCAGGATTCCTACAGG

AAGCAAGTAGTAATTGATGGAGAAACCTGTCTCTTGGATATTCTCGACACAGCAGGTCAAGAGGAGTACA

GTGCAATGAGGGACCAGTACATGAGGACTGGGGAGGGCTTTCTTTGTGTATTTGCCATAAATAATACTAA

ATCATTTGAAGATATTCACCATTATAGGTGGGTTTAAATTGAATATAATAAGCTGACATTAAGGAGTAAT

*KRAS* Exon 4

GAAATAAATGTGATTTGCCTTCTAGAACAGTAGACACAAAACAGGCTCAGGACTTAGCAAGAAGTTATGG

AATTCCTTTTATTGAAACATCAGCAAAGACAAGACAGGTAAGTAACACTGAAATAAATACAGATCTGTTT

TCTGCAAAATCATAACTGTTATGTCATTTAATATATCAGT
