# Supplementary material for: Development and Validation of a Clinical Trial Patient Stratification Assay That Interrogates 27 Mutation Sites in MAPK Pathway Genes
Source: PLoS One. 2013 Aug 21;8(8):e72239. doi: 10.1371/journal.pone.0072239 (PMC3749116; doi:10.1371/journal.pone.0072239)
Supplement: Figure S2 — Effect of PCR Primer Ratios on Amplification of DNA Fragments. The ability to amplify DNA fragments was dependent on the ratio of the PCR primer sets to each other. Increasing the concentration of one set of primers relative to the second set favored to amplification of one product over the other. The above exercise was repeated with the appropriate PCR primers until all PCR products were amplified to similar amounts as judged by gel analysis. (DOCX) [file pone.0072239.s002.docx]

Figure S2. Effect of PCR Primer Ratios on Amplification of DNA Fragments


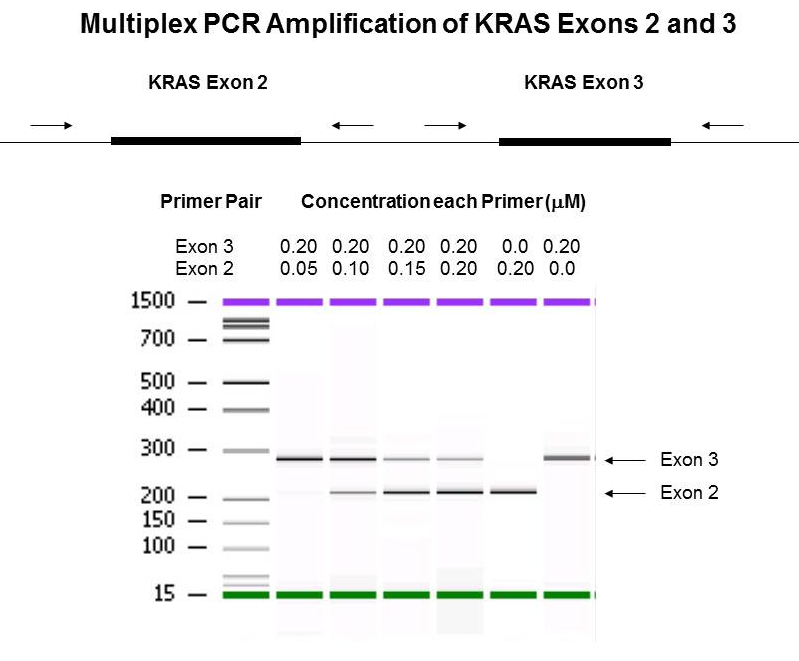


Figure S2 Legend. The ability to amplify DNA fragments was dependent on the ratio of the PCR primer sets to each other. Increasing the concentration of one set of primers relative to the second set favored to amplification of one product over the other. The above exercise was repeated with the appropriate PCR primers until all PCR products were amplified to similar amounts as judged by gel analysis.
