# Supplementary material for: Development and Validation of a Clinical Trial Patient Stratification Assay That Interrogates 27 Mutation Sites in MAPK Pathway Genes
Source: PLoS One. 2013 Aug 21;8(8):e72239. doi: 10.1371/journal.pone.0072239 (PMC3749116; doi:10.1371/journal.pone.0072239)
Supplement: Figure S3 — Failure of large DNA fragments to be amplified from FFPE derived gDNA in PCR. Large fragments of DNA fail to be amplified from FFPE derived gDNA. Replicate samples of FFPE gDNA from four tumor types (Ovarian cancer, lanes 1, 5, 9; Lung cancer, lanes 2, 6 10; colorectal cancer, lanes 3, 7, 11; pancreatic cancer, lanes 4, 8, 12) were subjected to PCR amplification for KRAS, NRAS and BRAF. PCR primers for KRAS were designed to amplify fragments of DNA 200–300 bp in size while PCR primers for NRAS and BRAF were designed to amplify fragments 125–150 bp in size. Three out of four PCR reactions for KRAS failed to successfully amplify DNA fragments while all PCR reactions of NRAS and BRAF were successful. (DOCX) [file pone.0072239.s003.docx]

Figure S3. Failure of large DNA fragments to be amplified from FFPE derived gDNA in PCR.


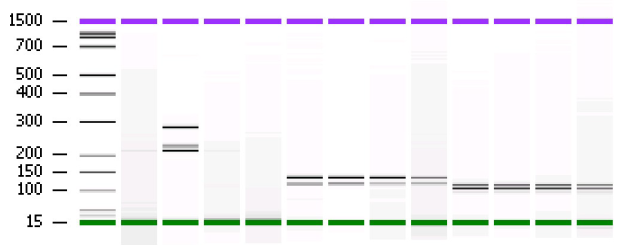


*KRAS*

*BRAF*

*NRAS*

(bp)

1

2

3

4

5

6

7

8

9

10

11

12


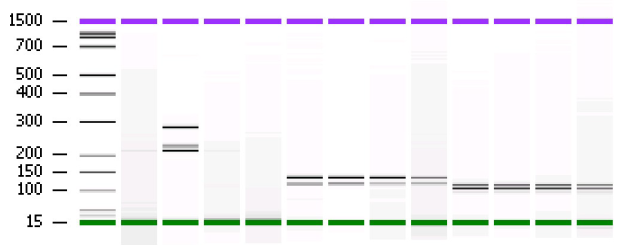


*KRAS*

*BRAF*

*NRAS*

(bp)

1

2

3

4

5

6

7

8

9

10

11

12

Figure S3 Legend. Large fragments of DNA fail to be amplified from FFPE derived gDNA. Replicate samples of FFPE gDNA from four tumor types (Ovarian cancer, lanes 1, 5, 9; Lung cancer, lanes 2, 6 10; colorectal cancer, lanes 3, 7, 11; pancreatic cancer, lanes 4, 8, 12) were subject to PCR amplification for *KRAS*, *NRAS* and *BRAF*. PCR primers for *KRAS* were designed to amplify fragments of DNA 200-300 bp in size while PCR primers for *NRAS* and *BRAF* were designed to amplify fragments 125-150 bp in size. Three out of four PCR reactions for *KRAS* failed to successfully amplify DNA fragments while all PCR reactions of *NRAS* and *BRAF* were successful.
