# Supplementary material for: Development and Validation of a Clinical Trial Patient Stratification Assay That Interrogates 27 Mutation Sites in MAPK Pathway Genes
Source: PLoS One. 2013 Aug 21;8(8):e72239. doi: 10.1371/journal.pone.0072239 (PMC3749116; doi:10.1371/journal.pone.0072239)
Supplement: Table S1 — Sequences and Working Stocks for PCR Primers and SNPE Reaction Probes. (DOCX) [file pone.0072239.s004.docx]

Table S1. Sequences and Working Stocks for PCR Primers and SNPE Reaction Probes.

**PCR Primers Sequences and Pools**

| **Gene** | **Region** | **Sequence (5' to 3')** | **Tm (^o^C)** | **Final Con. (nM)** |
| --- | --- | --- | --- | --- |
| ***KRAS* Pool** | exon 2, forward | TGTGACATGTTCTAATATAGTCACATT | 59.32 | 500 |
|  | exon 2, reverse | CACAAAATGATTCTGAATTAGCT | 58.8 | 500 |
|  | exon 3, forward | GGAAGCAAGTAGTAATTGATGG | 59.14 | 200 |
|  | exon 3, reverse | AAAGAAAGCCCTCCCC | 59.07 | 200 |
|  | exon 4, forward | GAACAGTAGACACAAAACAGGC | 60.23 | 200 |
|  | exon 4, reverse | TGCAGAAAACAGATCTGTATTTATTT | 60.73 | 200 |
| ***NRAS* Pool** | exon 2, forward | GGTGTGAAATGACTGAGTAC | 54.21 | 100 |
|  | exon 2, reverse | GGGCCTCACCTCTATGGTG | 64.49 | 100 |
|  | exon 3, forward | GGTGAAACCTGTTTGTTGGA | 62.38 | 150 |
|  | exon 3, reverse | ATACACAGAGGAAGCCTTCG | 60.82 | 150 |
| ***BRAF* Pool** | exon15, forward | TCTTCATGAAGACCTCACAGT | 58.85 | 100 |
|  | exon15, reverse | CCAGACAACTGTTCAAACTGA | 60.35 | 100 |
|  | exon 11, forward | GTGATGATTGGGAGATTCCT | 60.3 | 150 |
|  | exon 11, reverse | CTGCCACATCACCATGCCA | 69.62 | 150 |
