# Supplementary material for: Development and Validation of a Clinical Trial Patient Stratification Assay That Interrogates 27 Mutation Sites in MAPK Pathway Genes
Source: PLoS One. 2013 Aug 21;8(8):e72239. doi: 10.1371/journal.pone.0072239 (PMC3749116; doi:10.1371/journal.pone.0072239)
Supplement: Table S2 — SNPE Probe Sequences and Pools. (DOCX) [file pone.0072239.s005.docx]

**Table S2. SNPE Probe Sequences and Pools**

| **Reaction** | **Region** | **Sequence (5' to 3')** | **Size (nt)** | **Strand Probed** | **Tm (^o^C)*** | **Final Con. (nM)** |
| --- | --- | --- | --- | --- | --- | --- |
| **KRAS** | 34 / 12 | AACTTGTGGTAGTTGGAGCT | 20 | sense | 57.84 | 25 |
|  | 35 / 12 | GATCGTACTTGTGGTAGTTGGAGCTG | 26 | sense | 60.24 | 75 |
|  | 37 / 13 | GATCGATCGATCTTGTGGTAGTTGGAGCTGGT | 32 | sense | 62.15 | 25 |
|  | 38 / 13 | GATCGATCGATCGATCGATGTGGTAGTTGGAGCTGGTG | 38 | sense | 65.61 | 75 |
|  | 181 / 61 | T28 CTCATTGCACTGTACTCCTCTT | 50 | antisense | 59.95 | 25 |
|  | 182 / 61 | T35 ATTCTCGACACAGCAGGTC | 54 | sense | 60.45 | 25 |
|  | 183 / 61 | T42 CCTCATTGCACTGTACTCCTC | 63 | antisense | 61.29 | 65 |
|  | 436 / 146 | T49 GTCTTACTTAGCTGTCTTGTCTTTG | 73 | sense | 59.23 | 400 |
|  | 437 / 146 | T10 C10 T36 GAATTCCTTTTATTGAAACATCAG | 81 | antisense | 59.41 | 400 |
| **NRAS** | 35 / 12 | T22 CTGGTGGTGGTTGGAGCAG | 41 | sense | 66.93 | 75 |
|  | 37 / 13 | T28 GGTGGTGGTTGGAGCAGGT | 47 | sense | 67.46 | 3 |
|  | 38 / 13 | T32 GTCAGTGCGCTTTTCCCAACA | 53 | antisense | 69.39 | 125 |
|  | 181 / 61 | T37 CTCATGGCACTGTACTCTTCTT | 59 | antisense | 59.95 | 263 |
|  | 182 / 61 | T43 GACATACTGGATACAGCTGGAC | 65 | sense | 60.04 | 35 |
|  | 183 / 61 | T49 CTCTCATGGCACTGTACTCTTC | 71 | antisense | 60.48 | 70 |
| **BRAF-1** | 1774 / 592 | T15 CATGAAGACCTCACAGTAAAA | 36 | sense | 56.8 | 250 |
|  | 1781 / 594 | T21 ACCTCACAGTAAAAATAGGTG | 42 | sense | 54.84 | 250 |
|  | 1782 / 594 | T25 GATTTCACTGTAGCTAGACCAAA | 48 | antisense | 58.94 | 125 |
|  | 1786 / 596 | T33 GAGATTTCACTGTAGCTAGAC | 54 | antisense | 52.16 | 250 |
|  | 1789 / 597 | T39 GTAAAAATAGGTGATTTTGGT | 60 | sense | 53.91 | 250 |
|  | 1790 / 597 | T45 CATCGAGATTTCACTGTAGCT | 66 | antisense | 58.52 | 250 |
| **BRAF-2** | 1799(A) / 600 | T13 GGTGATTTTGGTCTAGCTACAA | 35 | sense | 59.65 | 62 |
|  | 1799(G) / 600 | T19 GGTGATTTTGGTCTAGCTACAG | 41 | sense | 59.29 | 62 |
|  | 1800 / 600 | T26 GGACCCACTCCATCGAGATTT | 47 | antisense | 66.21 | 62 |
|  | 1798 / 600 | T32 GGTGATTTTGGTCTAGCTACA | 53 | sense | 58.28 | 31 |
|  | 1801 / 601 | T39 GGACCCACTCCATCGAGATT | 59 | antisense | 65.16 | 62 |
|  | 2/ 466 | T45 GGGACAAAGAATTGGATCTG | 65 | sense | 60.87 | 62 |
|  | 3 / 466 | T49 CCTTGTAGACTGTTCCAAATGA | 71 | antisense | 60.55 | 93 |
|  |  | * Poly T tails or GATC repeats were omitted for Tm calculations | |  |  |  |
