# Supplementary material for: Development and Validation of a Clinical Trial Patient Stratification Assay That Interrogates 27 Mutation Sites in MAPK Pathway Genes
Source: PLoS One. 2013 Aug 21;8(8):e72239. doi: 10.1371/journal.pone.0072239 (PMC3749116; doi:10.1371/journal.pone.0072239)
Supplement: Table S3 — Sequence for Synthetic Oligonucleotides. (DOCX) [file pone.0072239.s006.docx]

**Table S3. Sequence for Synthetic Oligonucleotides.**

|  | Codon Number | Codon | Protein Description | **Sequence** |
| --- | --- | --- | --- | --- |
| **BRAF** | 466 | GGA | G466G | TACAGTGGGACAAAGAATTGGATCT**GGA**TCATTTGGAACAGTCTACAAGGGAA |
|  | 466 | GGA | G466G | TTC CCT TGT AGA CTG TTC CAA ATG ATC CAG ATC CAA TTC TTT GTC CCA CTG TA |
|  | 466 | GAA | G466E | TTC CCT TGT AGA CTG TTC CAA ATG ATT CAG ATC CAA TTC TTT GTC CCA CTG TA |
|  | 466 | GTA | G466V | TACAGTGGGACAAAGAATTGGATCT**GTA**TCATTTGGAACAGTCTACAAGGGAA |
|  | 592 | ATA | I592I | TCA CTG TAG CTA GAC CAA AAT CAC CTA TTT TTA CTG TGA GGT CTT CAT GAA GA |
|  | 592 | GTA | I592V | TCA CTG TAG CTA GAC CAA AAT CAC CTA GTT TTA CTG TGA GGT CTT CAT GAA GA |
|  | 594 | GAT | D594D | TGAAGACCTCACAGTAAAAATAGGT**GAT**TTTGGTCTAGCTACAGTGAAATCTC |
|  | 594 | GAT | D594D | GAG ATT TCA CTG TAG CTA GAC CAA AAT CAC CTA TTT TTA CTG TGA GGT CTT CA |
|  | 594 | GTT | D594V | GAG ATT TCA CTG TAG CTA GAC CAA AAA CAC CTA TTT TTA CTG TGA GGT CTT CA |
|  | 594 | GAA | D594E | TGAAGACCTCACAGTAAAAATAGGT**GAA**TTTGGTCTAGCTACAGTGAAATCTC |
|  | 594 | GAG | D594E | TGAAGACCTCACAGTAAAAATAGGT**GAG**TTTGGTCTAGCTACAGTGAAATCTC |
|  | 596 | GGT | G596G | CCTCACAGTAAAAATAGGTGATTTT**GGT**CTAGCTACAGTGAAATCTCGATGGA |
|  | 596 | GGT | G596G | TCC ATC GAG ATT TCA CTG TAG CTA GAC CAA AAT CAC CTA TTT TTA CTG TGA GG |
|  | 596 | CGT | G596R | CCTCACAGTAAAAATAGGTGATTTT**CGT**CTAGCTACAGTGAAATCTCGATGGA |
|  | 597 | TCA | L597S | CCTCACAGTAAAAATAGGTGATTTTCGT**TCA**GCTACAGTGAAATCTCGATGGA |
|  | 597 | TCA | L597S | TCC ATC GAG ATT TCA CTG TAG CTG AAC GAA AAT CAC CTA TTT TTA CTG TGA GG |
|  | 600 | GTG | V600V | AATAGGTGATTTTGGTCTAGCTACA**GTG**AAATCTCGATGGAGTGGGTCCCATC |
|  | 600 | GTG | V600V | GAT GGG ACC CAC TCC ATC GAG ATT TCA CTG TAG CTA GAC CAA AAT CAC CTA TT |
|  | 600 | GAG | V600E | GAT GGG ACC CAC TCC ATC GAG ATT TCT CTG TAG CTA GAC CAA AAT CAC CTA TT |
|  | 600 | AGG | V600R | GAT GGG ACC CAC TCC ATC GAG ATT TCC TTG TAG CTA GAC CAA AAT CAC CTA TT |
|  | 600 | AAG | V600K | GAT GGG ACC CAC TCC ATC GAG ATT TCT TTG TAG CTA GAC CAA AAT CAC CTA TT |
|  | 600 | GAT | V600D | AATAGGTGATTTTGGTCTAGCTACA**GAT**AAATCTCGATGGAGTGGGTCCCATC |
|  | 600 | GAT | V600D | GAT GGG ACC CAC TCC ATC GAG ATT TAT CTG TAG CTA GAC CAA AAT CAC CTA TT |
|  | 601 | GAA | K601E | AGGTGATTTTGGTCTAGCTACAGTG**GAA**TCTCGATGGAGTGGGTCCCATCAGT |
| **KRAS** | 12 | GGT | G12G | GTA TCG TCA AGG CAC TCT TGC CTA C**GC CAC C**AG CTC CAA CTA CCA CAA GTT TA |
|  | 12 | AGT | G12S | GTA TCG TCA AGG CAC TCT TGC CTA C**GC CAC T**AG CTC CAA CTA CCA CAA GTT TA |
|  | 12 | TGT | G12C | GTA TCG TCA AGG CAC TCT TGC CTA C**GC CAC A**AG CTC CAA CTA CCA CAA GTT TA |
|  | 12 | GAT | G12D | GTA TCG TCA AGG CAC TCT TGC CTA C**GC CAT C**AG CTC CAA CTA CCA CAA GTT TA |
|  | 12 | GCT | G12A | GTA TCG TCA AGG CAC TCT TGC CTA C**GC CAG C**AG CTC CAA CTA CCA CAA GTT TA |
|  | 12 | GTT | G12V | GTA TCG TCA AGG CAC TCT TGC CTA C**GC CAA C**AG CTC CAA CTA CCA CAA GTT TA |
|  | 13 | GGC | G13G | GTA TCG TCA AGG CAC TCT TGC CTA C**GC CAC C**AG CTC CAA CTA CCA CAA GTT TA |
|  | 13 | CGC | G13R | GTA TCG TCA AGG CAC TCT TGC CTA CGC GAC CAG CTC CAA CTA CCA CAA GTT TA |
|  | 13 | GAC | G13D | GTA TCG TCA AGG CAC TCT TGC CTA CGT CAC CAG CTC CAA CTA CCA CAA GTT TA |
|  | 61 | CAA | Q61Q | CTTGGATATTCTCGACACAGCAGGT**CAA**GAGGAGTACAGTGCAATGAGGGACC |
|  | 61 | CAA | Q61Q | GGT CCC TCA TTG CAC TGT ACT CCT CTT GAC CTG CTG TGT CGA GAA TAT CCA AG |
|  | 61 | AAA | Q61K | CTTGGATATTCTCGACACAGCAGGT**AAA**GAGGAGTACAGTGCAATGAGGGACC |
|  | 61 | CTA | Q61L | GGT CCC TCA TTG CAC TGT ACT CCT CTT TAC CTG CTG TGT CGA GAA TAT CCA AG |
|  | 61 | CAC | Q61H | CTTGGATATTCTCGACACAGCAGGT**CAC**GAGGAGTACAGTGCAATGAGGGACC |
|  | 61 | CAT | Q61H | CTTGGATATTCTCGACACAGCAGGT**CAT**GAGGAGTACAGTGCAATGAGGGACC |
|  | 146 | GCA | A146A | TGGAATTCCTTTTATTGAAACATCA**GCA**AAGACAAGACAGGTAAGTAACACTG |
|  | 146 | GCA | A146A | CAG TGT TAC TTA CCT GTC TTG TCT TTG CTG ATG TTT CAA TAA AAG GAA TTC CA |
|  | 146 | ACA | A146T | CAG TGT TAC TTA CCT GTC TTG TCT TTG TTG ATG TTT CAA TAA AAG GAA TTC CA |
|  | 146 | ACA | A146T | TGGAATTCCTTTTATTGAAACATCA**ACA**AAGACAAGACAGGTAAGTAACACTG |
|  | 146 | GTA | A146V | TGGAATTCCTTTTATTGAAACATCA**GTA**AAGACAAGACAGGTAAGTAACACTG |
|  | 146 | GTA | A146V | CAG TGT TAC TTA CCT GTC TTG TCT TTA CTG ATG TTT CAA TAA AAG GAA TTC CA |
| **NRAS** | 12 | GGT | G12G | CAAACTGGTGGTGGTTGGAGCA**GGT**GGTGTTGGGAAAAGCGCACTGACAATCC |
|  | 12 | GGT | G12G | GGA TTG TCA GTG CGC TTT TCC CAA CAC CAC CTG CTC CAA CCA CCA CCA GTT TG |
|  | 12 | GAT | G12D | CAAACTGGTGGTGGTTGGAGCA**GAT**GGTGTTGGGAAAAGCGCACTGACAATCC |
|  | 12 | GTT | G12V | CAAACTGGTGGTGGTTGGAGCA**GTT**GGTGTTGGGAAAAGCGCACTGACAATCC |
|  | 13 | GAT | G13D | CAAACTGGTGGTGGTTGGAGCAGGT**GAT**GTTGGGAAAAGCGCACTGACAATCC |
|  | 13 | CGT | G13R | GGA TTG TCA GTG CGC TTT TCC CAA CAC GAC CTG CTC CAA CCA CCA CCA GTT TG |
|  | 61 | CAA | Q61Q | GTTGGACATACTGGATACAGCTGGA**CAA**GAAGAGTACAGTGCCATGAGAGACC |
|  | 61 | CAA | Q61Q | GGT CTC TCA TGG CAC TGT ACT CTT CTT GTC CAG CTG TAT CCA GTA TGT CCA AC |
|  | 61 | AAA | Q61K | GTTGGACATACTGGATACAGCTGGA**AAA**GAAGAGTACAGTGCCATGAGAGACC |
|  | 61 | CGA | Q61R | GGT CTC TCA TGG CAC TGT ACT CTT CTC GTC CAG CTG TAT CCA GTA TGT CCA AC |
|  | 61 | CTA | Q61L | GGT CTC TCA TGG CAC TGT ACT CTT CTA GTC CAG CTG TAT CCA GTA TGT CCAAC |
|  | 61 | CAC | Q61H | GTTGGACATACTGGATACAGCTGGA**CAC**GAAGAGTACAGTGCCATGAGAGACC |
|  | 61 | CAT | Q61H | GTTGGACATACTGGATACAGCTGGA**CAT**GAAGAGTACAGTGCCATGAGAGACC |
